# Supplementary material for: The Systems Biology Research Tool: evolvable open-source software
Source: BMC Syst Biol. 2008 Jun 29;2:55. doi: 10.1186/1752-0509-2-55 (PMC2446383; doi:10.1186/1752-0509-2-55)
Supplement: Additional file 1 — SBRT Archive. An archive of the current version of the Systems Biology Research Tool. [file 1752-0509-2-55-S1.zip › sbrt-1.4.0/doc/developers_guide/api/sbrt/kernel/util/class-use/UnmodifiableSet.html]

Uses of Class sbrt.kernel.util.UnmodifiableSet


|  |  |  |
| --- | --- | --- |
| **Overview** \| **Package** \| **Class** \| **Use** \| **Tree** \| **Deprecated** \| **Index** \| **Help** | |  |
| PREV   NEXT | **FRAMES**    **NO FRAMES**     **All Classes** |  |


---


## **Uses of Class sbrt.kernel.util.UnmodifiableSet**

| Packages that use UnmodifiableSet | |
| --- | --- |
| **sbrt.kernel.fba.proc** | Provides classes and interfaces for executing Flux Balance Analysis processes. |
| **sbrt.kernel.math.algebra** | Provides classes and interfaces for creating objects relevant to algebra. |
| **sbrt.kernel.math.geom** | Provides classes and interfaces for creating objects relevant to computational geometry. |
| **sbrt.kernel.math.geom.comp** | Provides classes and interfaces for performing geometry-related computations. |
| **sbrt.kernel.util** | Provides classes and interfaces for performing utility functions. |
| **sbrt.shell.io** | Provides classes and interfaces for reading and writing files. |
| **sbrt.shell.io.fba** | Provides classes and interfaces for reading and writing files relevant to Flux Balance Analysis. |
| **sbrt.shell.mng** | Provides classes and interfaces for creating process managers. |
| **sbrt.shell.mng.algebra** | Provides classes for creating algebra process managers. |
| **sbrt.shell.mng.comb** | Provides classes for creating combinatorics process managers. |
| **sbrt.shell.mng.fba** | Provides classes and interfaces for creating Flux Balance Analysis process managers. |
| **sbrt.shell.mng.geom** | Provides classes and for creating geometry process managers. |
| **sbrt.shell.mng.graph\_theory** | Provides classes and for creating graph theoretical process managers. |
| **sbrt.shell.mng.stat** | Provides classes and interfaces for creating statistical process managers. |
| **sbrt.shell.mng.utilities** | Provides classes and interfaces for creating mathematical process managers. |

Uses of UnmodifiableSet in sbrt.kernel.fba.proc

| Methods in sbrt.kernel.fba.proc that return UnmodifiableSet | |
| --- | --- |
| `UnmodifiableSet<java.lang.String>` | `FluxPlasticityProcess.Results.getAlwaysActive()`             Returns the set of fluxes that are always active. |
| `UnmodifiableSet<java.lang.String>` | `FluxPlasticityProcess.Results.getNeverActive()`             Returns the set of fluxes that are never active. |
| `UnmodifiableSet<java.lang.String>` | `FluxPlasticityProcess.Results.getPotActive()`             Returns the set of fluxes that are potentially active. |

Uses of UnmodifiableSet in sbrt.kernel.math.algebra

| Methods in sbrt.kernel.math.algebra that return UnmodifiableSet | |
| --- | --- |
| `UnmodifiableSet<C>` | `MatrixWrap.getColumnIndices()`             Returns the set of column indices contained in this matrix. |
| `UnmodifiableSet<R>` | `MatrixWrap.getRowIndices()`             Returns the set of row indices contained in this matrix. |

Uses of UnmodifiableSet in sbrt.kernel.math.geom

| Methods in sbrt.kernel.math.geom that return UnmodifiableSet | |
| --- | --- |
| `UnmodifiableSet<ParallelHyperplanes<V>>` | `HashConvexPolytope.getBoundingPlanes()`             Returns the set of bounding hyperplanes that define this convex polytope. |
| `UnmodifiableSet<V>` | `HashConvexPolytope.getVariables()`             Returns the variables contained in the hyperplanes defining the bounding half-spaces. |

Uses of UnmodifiableSet in sbrt.kernel.math.geom.comp

| Methods in sbrt.kernel.math.geom.comp that return UnmodifiableSet | |
| --- | --- |
| `UnmodifiableSet<V>` | `CdHarComputer.Results.getUnchangedVariables()`             Returns the variables whose values are the same as they were in the previous point. |

Uses of UnmodifiableSet in sbrt.kernel.util

| Methods in sbrt.kernel.util that return UnmodifiableSet | |
| --- | --- |
| `UnmodifiableSet<java.util.Map.Entry<K,V>>` | `UnmodifiableMap.entrySet()`             See Map.entrySet(). |
| `UnmodifiableSet<E>` | `BooleanRelation.getElements()` |
| `UnmodifiableSet<K>` | `UnmodifiableMap.keySet()`             See Map.keySet(). |

Uses of UnmodifiableSet in sbrt.shell.io

| Methods in sbrt.shell.io that return UnmodifiableSet | |
| --- | --- |
| `UnmodifiableSet<K>` | `AbstractMapOutputFile.getFilterKeys()`             Returns the set of keys whose values will be written to this output file when a map is added. |
| `UnmodifiableSet<K>` | `RomiFile.getKeys()`             Returns the keys of the map contained in this file. |
| `UnmodifiableSet<K>` | `AbstractMapInputFile.getRequiredKeys()`             Returns the set of keys that must be contained in this file. |

Uses of UnmodifiableSet in sbrt.shell.io.fba

| Methods in sbrt.shell.io.fba that return UnmodifiableSet | |
| --- | --- |
| `UnmodifiableSet<E>` | `RxnNamExprSetInputFile.getRxnNameExprSet()`             Returns the set of reaction name expressions contained in this fle. |

Uses of UnmodifiableSet in sbrt.shell.mng

| Fields in sbrt.shell.mng declared as UnmodifiableSet | |
| --- | --- |
| `static UnmodifiableSet<java.lang.String>` | `AbstractProcessManager.optionalKeywords`             The set of optional keywords in the `ManagerInput` objects supplied to instances of this class. |
| `static UnmodifiableSet<java.lang.String>` | `AbstractProcessManager.requiredKeywords`             The set of keywords required to be present in the `ManagerInput` objects supplied to instances of this class. |

Uses of UnmodifiableSet in sbrt.shell.mng.algebra

| Fields in sbrt.shell.mng.algebra declared as UnmodifiableSet | |
| --- | --- |
| `static UnmodifiableSet<java.lang.String>` | `LinearSolverManager.optionalKeywords`             The set of keywords understood, but not required, by this process manager. |
| `static UnmodifiableSet<java.lang.String>` | `MultipleVectorsConverterManager.optionalKeywords`             The set of keywords understood, but not required, by this process manager. |
| `static UnmodifiableSet<java.lang.String>` | `MatrixConverterManager.optionalKeywords`             The set of keywords understood, but not required, by this process manager. |
| `static UnmodifiableSet<java.lang.String>` | `LinearCombConverterManager.optionalKeywords`             The set of keywords understood, but not required, by this process manager. |
| `static UnmodifiableSet<java.lang.String>` | `SingleVectorConverterManager.optionalKeywords`             The set of keywords understood, but not required, by this process manager. |
| `static UnmodifiableSet<java.lang.String>` | `LinearSolverManager.requiredKeywords`             The set of keywords required by this process manager. |
| `static UnmodifiableSet<java.lang.String>` | `MultipleVectorsConverterManager.requiredKeywords`             The set of keywords required by this process manager. |
| `static UnmodifiableSet<java.lang.String>` | `MatrixConverterManager.requiredKeywords`             The set of keywords required by this process manager. |
| `static UnmodifiableSet<java.lang.String>` | `LinearCombConverterManager.requiredKeywords`             The set of keywords required by this process manager. |
| `static UnmodifiableSet<java.lang.String>` | `SingleVectorConverterManager.requiredKeywords`             The set of keywords required by this process manager. |

| Methods in sbrt.shell.mng.algebra that return UnmodifiableSet | |
| --- | --- |
| `UnmodifiableSet<java.lang.String>` | `LinearSolverManager.getOptionalKeys()`             Returns the set of keywords understood, but not required, by this process manager. |
| `UnmodifiableSet<java.lang.String>` | `MultipleVectorsConverterManager.getOptionalKeys()`             Returns the set of keywords understood, but not required, by this process manager. |
| `UnmodifiableSet<java.lang.String>` | `MatrixConverterManager.getOptionalKeys()`             Returns the set of keywords understood, but not required, by this process manager. |
| `UnmodifiableSet<java.lang.String>` | `LinearCombConverterManager.getOptionalKeys()`             Returns the set of keywords understood, but not required, by this process manager. |
| `UnmodifiableSet<java.lang.String>` | `SingleVectorConverterManager.getOptionalKeys()`             Returns the set of keywords understood, but not required, by this process manager. |
| `UnmodifiableSet<java.lang.String>` | `LinearSolverManager.getRequiredKeys()`             Returns the set of keywords required by this process manager. |
| `UnmodifiableSet<java.lang.String>` | `MultipleVectorsConverterManager.getRequiredKeys()`             Returns the set of keywords required by this process manager. |
| `UnmodifiableSet<java.lang.String>` | `MatrixConverterManager.getRequiredKeys()`             Returns the set of keywords required by this process manager. |
| `UnmodifiableSet<java.lang.String>` | `LinearCombConverterManager.getRequiredKeys()`             Returns the set of keywords required by this process manager. |
| `UnmodifiableSet<java.lang.String>` | `SingleVectorConverterManager.getRequiredKeys()`             Returns the set of keywords required by this process manager. |

Uses of UnmodifiableSet in sbrt.shell.mng.comb

| Fields in sbrt.shell.mng.comb declared as UnmodifiableSet | |
| --- | --- |
| `static UnmodifiableSet<java.lang.String>` | `StrictSingleElementUnionManager.optionalKeywords`             The set of keywords understood, but not required, by this process manager. |
| `static UnmodifiableSet<java.lang.String>` | `SingleElementUnionManager.optionalKeywords`             The set of keywords understood, but not required, by this process manager. |
| `static UnmodifiableSet<java.lang.String>` | `StrictSingleElementUnionManager.requiredKeywords`             The set of keywords required by this process manager. |
| `static UnmodifiableSet<java.lang.String>` | `SingleElementUnionManager.requiredKeywords`             The set of keywords required by this process manager. |

| Methods in sbrt.shell.mng.comb that return UnmodifiableSet | |
| --- | --- |
| `UnmodifiableSet<java.lang.String>` | `StrictSingleElementUnionManager.getOptionalKeys()`             Returns the set of keywords understood, but not required, by this process manager. |
| `UnmodifiableSet<java.lang.String>` | `SingleElementUnionManager.getOptionalKeys()`             Returns the set of keywords understood, but not required, by this process manager. |
| `UnmodifiableSet<java.lang.String>` | `StrictSingleElementUnionManager.getRequiredKeys()`             Returns the set of keywords required by this process manager. |
| `UnmodifiableSet<java.lang.String>` | `SingleElementUnionManager.getRequiredKeys()`             Returns the set of keywords required by this process manager. |

Uses of UnmodifiableSet in sbrt.shell.mng.fba

| Fields in sbrt.shell.mng.fba declared as UnmodifiableSet | |
| --- | --- |
| `static UnmodifiableSet<java.lang.String>` | `WwFluxomeReducerManager.optionalKeywords`             The set of keywords understood, but not required, by instances of this class. |
| `static UnmodifiableSet<java.lang.String>` | `RandomConstraintsManager.optionalKeywords`             The set of keywords understood, but not required, by instances of this class. |
| `static UnmodifiableSet<java.lang.String>` | `CdHarFluxVectorManager.optionalKeywords`             The set of keywords required to be present in the `ManagerInput` objects supplied to instances of this class. |
| `static UnmodifiableSet<java.lang.String>` | `RandomEnvManager.optionalKeywords`             The set of keywords understood, but not required, by instances of this class. |
| `static UnmodifiableSet<java.lang.String>` | `ExtremeCurrentManager.optionalKeywords`             The set of optional keywords in the `ManagerInput` objects supplied to instances of this class. |
| `static UnmodifiableSet<java.lang.String>` | `CvFbaOptManager.optionalKeywords`             The set of keywords understood, but not required, by instances of this class. |
| `static UnmodifiableSet<java.lang.String>` | `BiggSbmlTranslaterManager.optionalKeywords`             The set of keywords understood, but not required, by this process manager. |
| `static UnmodifiableSet<java.lang.String>` | `NetworkInfoManager.optionalKeywords`             The set of keywords understood, but not required, by instances of this class. |
| `static UnmodifiableSet<java.lang.String>` | `StoichVariationManager.optionalKeywords`             The set of keywords understood, but not required, by instances of this class. |
| `static UnmodifiableSet<java.lang.String>` | `RxnRemovalManager.optionalKeywords`             The set of keywords understood, but not required, by instances of this class. |
| `static UnmodifiableSet<java.lang.String>` | `FluxCapManager.optionalKeywords`             The set of keywords understood, but not required, by instances of this class. |
| `static UnmodifiableSet<java.lang.String>` | `ReverseRxnFluxIntervalManager.optionalKeywords`             The set of keywords understood, but not required, by instances of this class. |
| `static UnmodifiableSet<java.lang.String>` | `SimpleFluxIntervalManager.optionalKeywords`             The set of keywords understood, but not required, by instances of this class. |
| `static UnmodifiableSet<java.lang.String>` | `FbaOptManager.optionalKeywords`             The set of keywords understood, but not required, by instances of this class. |
| `static UnmodifiableSet<java.lang.String>` | `RxnDeletionManager.optionalKeywords`             The set of optional keywords in the `ManagerInput` objects supplied to instances of this class. |
| `static UnmodifiableSet<java.lang.String>` | `CvRxnDeletionManager.optionalKeywords`             The set of keywords understood, but not required, by instances of this class. |
| `static UnmodifiableSet<java.lang.String>` | `ObjectiveFunctionManager.optionalKeywords`             The set of keywords understood, but not required, by instances of this class. |
| `static UnmodifiableSet<java.lang.String>` | `SlpCycleIdManager.optionalKeywords`             The set of keywords understood, but not required, by instances of this class. |
| `static UnmodifiableSet<java.lang.String>` | `SimpleRxnFileTransManager.optionalKeywords`             The set of keywords understood, but not required, by this process manager. |
| `static UnmodifiableSet<java.lang.String>` | `SvInitialPointManager.optionalKeywords`             The set of optional keywords in the `ManagerInput` objects supplied to instances of this class. |
| `static UnmodifiableSet<java.lang.String>` | `MetatoolFileWriterManager.optionalKeywords`             The set of keywords understood, but not required, by this process manager. |
| `static UnmodifiableSet<java.lang.String>` | `SbmlWriterManager.optionalKeywords`             The set of keywords understood, but not required, by this process manager. |
| `static UnmodifiableSet<java.lang.String>` | `FbaManager.optionalKeywords`             The set of keywords understood, but not required, by instances of this class. |
| `static UnmodifiableSet<java.lang.String>` | `CvCatalystDeletionManager.optionalKeywords`             The set of keywords required to be present in the `ManagerInput` objects supplied to instances of this class. |
| `static UnmodifiableSet<java.lang.String>` | `MsFluxomeReducerManager.optionalKeywords`             The set of keywords understood, but not required, by instances of this class. |
| `static UnmodifiableSet<java.lang.String>` | `FbaSolverManager.optionalKeywords`             The set of keywords understood, but not required, by instances of this class. |
| `static UnmodifiableSet<java.lang.String>` | `MmaNotebookManager.optionalKeywords`             The set of keywords understood, but not required, by instances of this class. |
| `static UnmodifiableSet<java.lang.String>` | `BiggSbmlReaderManager.optionalKeywords`             The set of keywords understood, but not required, by this process manager. |
| `static UnmodifiableSet<java.lang.String>` | `CvRevRxnFluxIntervalManager.optionalKeywords`             The set of keywords understood, but not required, by instances of this class. |
| `static UnmodifiableSet<java.lang.String>` | `FluxPlasticityManager.optionalKeywords`             The set of keywords understood, but not required, by instances of this class. |
| `static UnmodifiableSet<java.lang.String>` | `MsFluxIntervalManager.optionalKeywords`             The set of keywords understood, but not required, by instances of this class. |
| `static UnmodifiableSet<java.lang.String>` | `RandomObjectiveManager.optionalKeywords`             The set of keywords understood, but not required, by instances of this class. |
| `static UnmodifiableSet<java.lang.String>` | `CvMsFluxIntervalManager.optionalKeywords`             The set of keywords understood, but not required, by instances of this class. |
| `static UnmodifiableSet<java.lang.String>` | `PalssonSbmlTranslaterManager.optionalKeywords`             The set of keywords understood, but not required, by this process manager. |
| `static UnmodifiableSet<java.lang.String>` | `SvCdHarFluxVectorManager.optionalKeywords`             The set of keywords required to be present in the `ManagerInput` objects supplied to instances of this class. |
| `static UnmodifiableSet<java.lang.String>` | `CvSimpleFluxIntervalManager.optionalKeywords`             The set of keywords understood, but not required, by instances of this class. |
| `static UnmodifiableSet<java.lang.String>` | `CatalystDeletionManager.optionalKeywords`             The set of optional keywords in the `ManagerInput` objects supplied to instances of this class. |
| `static UnmodifiableSet<java.lang.String>` | `InitialPointManager.optionalKeywords`             The set of keywords understood, but not required, by instances of this class. |
| `static UnmodifiableSet<java.lang.String>` | `PalssonSbmlReaderManager.optionalKeywords`             The set of keywords understood, but not required, by this process manager. |
| `static UnmodifiableSet<java.lang.String>` | `CvObjectiveFunctionManager.optionalKeywords`             The set of optional keywords in the `ManagerInput` objects supplied to instances of this class. |
| `static UnmodifiableSet<java.lang.String>` | `FluxActivityManager.optionalKeywords`             The set of optional keywords in the `ManagerInput` objects supplied to instances of this class. |
| `static UnmodifiableSet<java.lang.String>` | `WwFluxomeReducerManager.requiredKeywords`             The set of keywords required to be present in the `ManagerInput` objects supplied to instances of this class. |
| `static UnmodifiableSet<java.lang.String>` | `RandomConstraintsManager.requiredKeywords`             The set of keywords required to be present in the `ManagerInput` objects supplied to instances of this class. |
| `static UnmodifiableSet<java.lang.String>` | `CdHarFluxVectorManager.requiredKeywords`             The set of keywords required to be present in the `ManagerInput` objects supplied to instances of this class. |
| `static UnmodifiableSet<java.lang.String>` | `RandomEnvManager.requiredKeywords`             The set of keywords required to be present in the `ManagerInput` objects supplied to instances of this class. |
| `static UnmodifiableSet<java.lang.String>` | `ExtremeCurrentManager.requiredKeywords`             The set of keywords required to be present in the `ManagerInput` objects supplied to instances of this class. |
| `static UnmodifiableSet<java.lang.String>` | `CvFbaOptManager.requiredKeywords`             The set of keywords required to be present in the `ManagerInput` objects supplied to instances of this class. |
| `static UnmodifiableSet<java.lang.String>` | `BiggSbmlTranslaterManager.requiredKeywords`             The set of keywords required by this process manager. |
| `static UnmodifiableSet<java.lang.String>` | `NetworkInfoManager.requiredKeywords`             The set of keywords required to be present in the `ManagerInput` objects supplied to instances of this class. |
| `static UnmodifiableSet<java.lang.String>` | `StoichVariationManager.requiredKeywords`             The set of keywords required to be present in the `ManagerInput` objects supplied to instances of this class. |
| `static UnmodifiableSet<java.lang.String>` | `RxnRemovalManager.requiredKeywords`             The set of keywords required to be present in the `ManagerInput` objects supplied to instances of this class. |
| `static UnmodifiableSet<java.lang.String>` | `FluxCapManager.requiredKeywords`             The set of keywords required to be present in the `ManagerInput` objects supplied to instances of this class. |
| `static UnmodifiableSet<java.lang.String>` | `ReverseRxnFluxIntervalManager.requiredKeywords`             The set of keywords required to be present in the `ManagerInput` objects supplied to instances of this class. |
| `static UnmodifiableSet<java.lang.String>` | `SimpleFluxIntervalManager.requiredKeywords`             The set of keywords required to be present in the `ManagerInput` objects supplied to instances of this class. |
| `static UnmodifiableSet<java.lang.String>` | `FbaOptManager.requiredKeywords`             The set of keywords required to be present in the `ManagerInput` objects supplied to instances of this class. |
| `static UnmodifiableSet<java.lang.String>` | `RxnDeletionManager.requiredKeywords`             The set of keywords required to be present in the `ManagerInput` objects supplied to instances of this class. |
| `static UnmodifiableSet<java.lang.String>` | `CvRxnDeletionManager.requiredKeywords`             The set of keywords required to be present in the `ManagerInput` objects supplied to instances of this class. |
| `static UnmodifiableSet<java.lang.String>` | `ObjectiveFunctionManager.requiredKeywords`             The set of keywords required to be present in the `ManagerInput` objects supplied to instances of this class. |
| `static UnmodifiableSet<java.lang.String>` | `SlpCycleIdManager.requiredKeywords`             The set of keywords required to be present in the `ManagerInput` objects supplied to instances of this class. |
| `static UnmodifiableSet<java.lang.String>` | `SimpleRxnFileTransManager.requiredKeywords`             The set of keywords required by this process manager. |
| `static UnmodifiableSet<java.lang.String>` | `SvInitialPointManager.requiredKeywords`             The set of keywords required to be present in the `ManagerInput` objects supplied to instances of this class. |
| `static UnmodifiableSet<java.lang.String>` | `MetatoolFileWriterManager.requiredKeywords`             The set of keywords required by this process manager. |
| `static UnmodifiableSet<java.lang.String>` | `SbmlWriterManager.requiredKeywords`             The set of keywords required by this process manager. |
| `static UnmodifiableSet<java.lang.String>` | `FbaManager.requiredKeywords`             The set of keywords required to be present in the `ManagerInput` objects supplied to instances of this class. |
| `static UnmodifiableSet<java.lang.String>` | `CvCatalystDeletionManager.requiredKeywords`             The set of keywords required to be present in the `ManagerInput` objects supplied to instances of this class. |
| `static UnmodifiableSet<java.lang.String>` | `MsFluxomeReducerManager.requiredKeywords`             The set of keywords required to be present in the `ManagerInput` objects supplied to instances of this class. |
| `static UnmodifiableSet<java.lang.String>` | `FbaSolverManager.requiredKeywords`             The set of keywords required to be present in the `ManagerInput` objects supplied to instances of this class. |
| `static UnmodifiableSet<java.lang.String>` | `MmaNotebookManager.requiredKeywords`             The set of keywords required to be present in the `ManagerInput` objects supplied to instances of this class. |
| `static UnmodifiableSet<java.lang.String>` | `BiggSbmlReaderManager.requiredKeywords`             The set of keywords required by this process manager. |
| `static UnmodifiableSet<java.lang.String>` | `CvRevRxnFluxIntervalManager.requiredKeywords`             The set of keywords required to be present in the `ManagerInput` objects supplied to instances of this class. |
| `static UnmodifiableSet<java.lang.String>` | `FluxPlasticityManager.requiredKeywords`             The set of keywords required to be present in the `ManagerInput` objects supplied to instances of this class. |
| `static UnmodifiableSet<java.lang.String>` | `MsFluxIntervalManager.requiredKeywords`             The set of keywords required to be present in the `ManagerInput` objects supplied to instances of this class. |
| `static UnmodifiableSet<java.lang.String>` | `RandomObjectiveManager.requiredKeywords`             The set of keywords required to be present in the `ManagerInput` objects supplied to instances of this class. |
| `static UnmodifiableSet<java.lang.String>` | `CvMsFluxIntervalManager.requiredKeywords`             The set of keywords required to be present in the `ManagerInput` objects supplied to instances of this class. |
| `static UnmodifiableSet<java.lang.String>` | `PalssonSbmlTranslaterManager.requiredKeywords`             The set of keywords required by this process manager. |
| `static UnmodifiableSet<java.lang.String>` | `SvCdHarFluxVectorManager.requiredKeywords`             The set of keywords required to be present in the `ManagerInput` objects supplied to instances of this class. |
| `static UnmodifiableSet<java.lang.String>` | `CvSimpleFluxIntervalManager.requiredKeywords`             The set of keywords required to be present in the `ManagerInput` objects supplied to instances of this class. |
| `static UnmodifiableSet<java.lang.String>` | `CatalystDeletionManager.requiredKeywords`             The set of keywords required to be present in the `ManagerInput` objects supplied to instances of this class. |
| `static UnmodifiableSet<java.lang.String>` | `InitialPointManager.requiredKeywords`             The set of keywords required to be present in the `ManagerInput` objects supplied to instances of this class. |
| `static UnmodifiableSet<java.lang.String>` | `PalssonSbmlReaderManager.requiredKeywords`             The set of keywords required by this process manager. |
| `static UnmodifiableSet<java.lang.String>` | `CvObjectiveFunctionManager.requiredKeywords`             The set of keywords required to be present in the `ManagerInput` objects supplied to instances of this class. |
| `static UnmodifiableSet<java.lang.String>` | `FluxActivityManager.requiredKeywords`             The set of keywords required to be present in the `ManagerInput` objects supplied to instances of this class. |

| Methods in sbrt.shell.mng.fba that return UnmodifiableSet | |
| --- | --- |
| `UnmodifiableSet<java.lang.String>` | `WwFluxomeReducerManager.getOptionalKeys()`             Returns the set of keywords the provided `ManagerInput` object does not necessarily have to contain. |
| `UnmodifiableSet<java.lang.String>` | `RandomConstraintsManager.getOptionalKeys()`             Returns the set of keywords the provided `ManagerInput` object does not necessarily have to contain. |
| `UnmodifiableSet<java.lang.String>` | `CdHarFluxVectorManager.getOptionalKeys()`             Returns the set of keywords the provided `ManagerInput` object does not necessarily have to contain. |
| `UnmodifiableSet<java.lang.String>` | `RandomEnvManager.getOptionalKeys()`             Returns the set of keywords the provided `ManagerInput` object does not necessarily have to contain. |
| `UnmodifiableSet<java.lang.String>` | `ExtremeCurrentManager.getOptionalKeys()`             Returns the set of keywords the provided `ManagerInput` object does not necessarily have to contain. |
| `UnmodifiableSet<java.lang.String>` | `CvFbaOptManager.getOptionalKeys()`             Returns the set of keywords the provided `ManagerInput` object does not necessarily have to contain. |
| `UnmodifiableSet<java.lang.String>` | `BiggSbmlTranslaterManager.getOptionalKeys()`             Returns the set of keywords understood, but not required, by this process manager. |
| `UnmodifiableSet<java.lang.String>` | `NetworkInfoManager.getOptionalKeys()`             Returns the set of keywords the provided `ManagerInput` object does not necessarily have to contain. |
| `UnmodifiableSet<java.lang.String>` | `StoichVariationManager.getOptionalKeys()`             Returns the set of keywords the provided `ManagerInput` object does not necessarily have to contain. |
| `UnmodifiableSet<java.lang.String>` | `RxnRemovalManager.getOptionalKeys()`             Returns the set of keywords the provided `ManagerInput` object does not necessarily have to contain. |
| `UnmodifiableSet<java.lang.String>` | `FluxCapManager.getOptionalKeys()`             Returns the set of keywords the provided `ManagerInput` object does not necessarily have to contain. |
| `UnmodifiableSet<java.lang.String>` | `ReverseRxnFluxIntervalManager.getOptionalKeys()`             Returns the set of keywords the provided `ManagerInput` object does not necessarily have to contain. |
| `UnmodifiableSet<java.lang.String>` | `SimpleFluxIntervalManager.getOptionalKeys()`             Returns the set of keywords the provided `ManagerInput` object does not necessarily have to contain. |
| `UnmodifiableSet<java.lang.String>` | `FbaOptManager.getOptionalKeys()`             Returns the set of keywords the provided `ManagerInput` object does not necessarily have to contain. |
| `UnmodifiableSet<java.lang.String>` | `RxnDeletionManager.getOptionalKeys()`             Returns the set of keywords the provided `ManagerInput` object does not necessarily have to contain. |
| `UnmodifiableSet<java.lang.String>` | `ObjectiveFunctionManager.getOptionalKeys()`             Returns the set of keywords the provided `ManagerInput` object does not necessarily have to contain. |
| `UnmodifiableSet<java.lang.String>` | `SlpCycleIdManager.getOptionalKeys()`             Returns the set of keywords the provided `ManagerInput` object does not necessarily have to contain. |
| `UnmodifiableSet<java.lang.String>` | `SimpleRxnFileTransManager.getOptionalKeys()`             Returns the set of keywords understood, but not required, by this process manager. |
| `UnmodifiableSet<java.lang.String>` | `SvInitialPointManager.getOptionalKeys()`             Returns the set of keywords the provided `ManagerInput` object does not necessarily have to contain. |
| `UnmodifiableSet<java.lang.String>` | `MetatoolFileWriterManager.getOptionalKeys()`             Returns the set of keywords understood, but not required, by this process manager. |
| `UnmodifiableSet<java.lang.String>` | `SbmlWriterManager.getOptionalKeys()`             Returns the set of keywords understood, but not required, by this process manager. |
| `UnmodifiableSet<java.lang.String>` | `CvCatalystDeletionManager.getOptionalKeys()`             Returns the set of keywords the provided `ManagerInput` object does not necessarily have to contain. |
| `UnmodifiableSet<java.lang.String>` | `MsFluxomeReducerManager.getOptionalKeys()`             Returns the set of keywords the provided `ManagerInput` object does not necessarily have to contain. |
| `UnmodifiableSet<java.lang.String>` | `FbaSolverManager.getOptionalKeys()`             Returns the set of keywords the provided `ManagerInput` object does not necessarily have to contain. |
| `UnmodifiableSet<java.lang.String>` | `MmaNotebookManager.getOptionalKeys()`             Returns the set of keywords the provided `ManagerInput` object does not necessarily have to contain. |
| `UnmodifiableSet<java.lang.String>` | `BiggSbmlReaderManager.getOptionalKeys()`             Returns the set of keywords understood, but not required, by this process manager. |
| `UnmodifiableSet<java.lang.String>` | `CvRevRxnFluxIntervalManager.getOptionalKeys()`             Returns the set of keywords the provided `ManagerInput` object does not necessarily have to contain. |
| `UnmodifiableSet<java.lang.String>` | `FluxPlasticityManager.getOptionalKeys()`             Returns the set of keywords the provided `ManagerInput` object does not necessarily have to contain. |
| `UnmodifiableSet<java.lang.String>` | `MsFluxIntervalManager.getOptionalKeys()`             Returns the set of keywords the provided `ManagerInput` object does not necessarily have to contain. |
| `UnmodifiableSet<java.lang.String>` | `RandomObjectiveManager.getOptionalKeys()`             Returns the set of keywords the provided `ManagerInput` object does not necessarily have to contain. |
| `UnmodifiableSet<java.lang.String>` | `CvMsFluxIntervalManager.getOptionalKeys()`             Returns the set of keywords the provided `ManagerInput` object does not necessarily have to contain. |
| `UnmodifiableSet<java.lang.String>` | `PalssonSbmlTranslaterManager.getOptionalKeys()`             Returns the set of keywords understood, but not required, by this process manager. |
| `UnmodifiableSet<java.lang.String>` | `SvCdHarFluxVectorManager.getOptionalKeys()`             Returns the set of keywords the provided `ManagerInput` object does not necessarily have to contain. |
| `UnmodifiableSet<java.lang.String>` | `CvSimpleFluxIntervalManager.getOptionalKeys()`             Returns the set of keywords the provided `ManagerInput` object does not necessarily have to contain. |
| `UnmodifiableSet<java.lang.String>` | `InitialPointManager.getOptionalKeys()`             Returns the set of keywords the provided `ManagerInput` object does not necessarily have to contain. |
| `UnmodifiableSet<java.lang.String>` | `PalssonSbmlReaderManager.getOptionalKeys()`             Returns the set of keywords understood, but not required, by this process manager. |
| `UnmodifiableSet<java.lang.String>` | `FluxActivityManager.getOptionalKeys()`             Returns the set of keywords the provided `ManagerInput` object does not necessarily have to contain. |
| `UnmodifiableSet<java.lang.String>` | `WwFluxomeReducerManager.getRequiredKeys()`             Returns the set of keywords the provided `ManagerInput` object must contain. |
| `UnmodifiableSet<java.lang.String>` | `RandomConstraintsManager.getRequiredKeys()`             Returns the set of keywords the provided `ManagerInput` object must contain. |
| `UnmodifiableSet<java.lang.String>` | `CdHarFluxVectorManager.getRequiredKeys()`             Returns the set of keywords the provided `ManagerInput` object must contain. |
| `UnmodifiableSet<java.lang.String>` | `RandomEnvManager.getRequiredKeys()`             Returns the set of keywords the provided `ManagerInput` object must contain. |
| `UnmodifiableSet<java.lang.String>` | `ExtremeCurrentManager.getRequiredKeys()`             Returns the set of keywords the provided `ManagerInput` object must contain. |
| `UnmodifiableSet<java.lang.String>` | `CvFbaOptManager.getRequiredKeys()`             Returns the set of keywords the provided `ManagerInput` object must contain. |
| `UnmodifiableSet<java.lang.String>` | `BiggSbmlTranslaterManager.getRequiredKeys()`             Returns the set of keywords required by this process manager. |
| `UnmodifiableSet<java.lang.String>` | `NetworkInfoManager.getRequiredKeys()`             Returns the set of keywords the provided `ManagerInput` object must contain. |
| `UnmodifiableSet<java.lang.String>` | `StoichVariationManager.getRequiredKeys()`             Returns the set of keywords the provided `ManagerInput` object must contain. |
| `UnmodifiableSet<java.lang.String>` | `RxnRemovalManager.getRequiredKeys()`             Returns the set of keywords the provided `ManagerInput` object must contain. |
| `UnmodifiableSet<java.lang.String>` | `FluxCapManager.getRequiredKeys()`             Returns the set of keywords the provided `ManagerInput` object must contain. |
| `UnmodifiableSet<java.lang.String>` | `ReverseRxnFluxIntervalManager.getRequiredKeys()`             Returns the set of keywords the provided `ManagerInput` object must contain. |
| `UnmodifiableSet<java.lang.String>` | `SimpleFluxIntervalManager.getRequiredKeys()`             Returns the set of keywords the provided `ManagerInput` object must contain. |
| `UnmodifiableSet<java.lang.String>` | `FbaOptManager.getRequiredKeys()`             Returns the set of keywords the provided `ManagerInput` object must contain. |
| `UnmodifiableSet<java.lang.String>` | `RxnDeletionManager.getRequiredKeys()`             Returns the set of keywords the provided `ManagerInput` object must contain. |
| `UnmodifiableSet<java.lang.String>` | `ObjectiveFunctionManager.getRequiredKeys()`             Returns the set of keywords the provided `ManagerInput` object must contain. |
| `UnmodifiableSet<java.lang.String>` | `SlpCycleIdManager.getRequiredKeys()`             Returns the set of keywords the provided `ManagerInput` object must contain. |
| `UnmodifiableSet<java.lang.String>` | `SimpleRxnFileTransManager.getRequiredKeys()`             Returns the set of keywords required by this process manager. |
| `UnmodifiableSet<java.lang.String>` | `SvInitialPointManager.getRequiredKeys()`             Returns the set of keywords the provided `ManagerInput` object must contain. |
| `UnmodifiableSet<java.lang.String>` | `MetatoolFileWriterManager.getRequiredKeys()`             Returns the set of keywords required by this process manager. |
| `UnmodifiableSet<java.lang.String>` | `SbmlWriterManager.getRequiredKeys()`             Returns the set of keywords required by this process manager. |
| `UnmodifiableSet<java.lang.String>` | `CvCatalystDeletionManager.getRequiredKeys()`             Returns the set of keywords the provided `ManagerInput` object must contain. |
| `UnmodifiableSet<java.lang.String>` | `MsFluxomeReducerManager.getRequiredKeys()`             Returns the set of keywords the provided `ManagerInput` object must contain. |
| `UnmodifiableSet<java.lang.String>` | `FbaSolverManager.getRequiredKeys()`             Returns the set of keywords the provided `ManagerInput` object must contain. |
| `UnmodifiableSet<java.lang.String>` | `MmaNotebookManager.getRequiredKeys()`             Returns the set of keywords the provided `ManagerInput` object must contain. |
| `UnmodifiableSet<java.lang.String>` | `BiggSbmlReaderManager.getRequiredKeys()`             Returns the set of keywords required by this process manager. |
| `UnmodifiableSet<java.lang.String>` | `CvRevRxnFluxIntervalManager.getRequiredKeys()`             Returns the set of keywords the provided `ManagerInput` object must contain. |
| `UnmodifiableSet<java.lang.String>` | `FluxPlasticityManager.getRequiredKeys()`             Returns the set of keywords the provided `ManagerInput` object must contain. |
| `UnmodifiableSet<java.lang.String>` | `MsFluxIntervalManager.getRequiredKeys()`             Returns the set of keywords the provided `ManagerInput` object must contain. |
| `UnmodifiableSet<java.lang.String>` | `RandomObjectiveManager.getRequiredKeys()`             Returns the set of keywords the provided `ManagerInput` object must contain. |
| `UnmodifiableSet<java.lang.String>` | `CvMsFluxIntervalManager.getRequiredKeys()`             Returns the set of keywords the provided `ManagerInput` object must contain. |
| `UnmodifiableSet<java.lang.String>` | `PalssonSbmlTranslaterManager.getRequiredKeys()`             Returns the set of keywords required by this process manager. |
| `UnmodifiableSet<java.lang.String>` | `SvCdHarFluxVectorManager.getRequiredKeys()`             Returns the set of keywords the provided `ManagerInput` object must contain. |
| `UnmodifiableSet<java.lang.String>` | `CvSimpleFluxIntervalManager.getRequiredKeys()`             Returns the set of keywords the provided `ManagerInput` object must contain. |
| `UnmodifiableSet<java.lang.String>` | `InitialPointManager.getRequiredKeys()`             Returns the set of keywords the provided `ManagerInput` object must contain. |
| `UnmodifiableSet<java.lang.String>` | `PalssonSbmlReaderManager.getRequiredKeys()`             Returns the set of keywords required by this process manager. |
| `UnmodifiableSet<java.lang.String>` | `FluxActivityManager.getRequiredKeys()`             Returns the set of keywords the provided `ManagerInput` object must contain. |

Uses of UnmodifiableSet in sbrt.shell.mng.geom

| Fields in sbrt.shell.mng.geom declared as UnmodifiableSet | |
| --- | --- |
| `static UnmodifiableSet<java.lang.String>` | `CdHarManager.optionalKeywords`             The set of keywords understood, but not required, by this process manager. |
| `static UnmodifiableSet<java.lang.String>` | `CdHarManager.requiredKeywords`             The set of keywords required by this process manager. |

| Methods in sbrt.shell.mng.geom that return UnmodifiableSet | |
| --- | --- |
| `UnmodifiableSet<java.lang.String>` | `CdHarManager.getOptionalKeys()`             Returns the set of keywords understood, but not required, by this process manager. |
| `UnmodifiableSet<java.lang.String>` | `CdHarManager.getRequiredKeys()`             Returns the set of keywords required by this process manager. |

Uses of UnmodifiableSet in sbrt.shell.mng.graph\_theory

| Fields in sbrt.shell.mng.graph\_theory declared as UnmodifiableSet | |
| --- | --- |
| `static UnmodifiableSet<java.lang.String>` | `CycleIdManager.optionalKeywords`             The set of keywords understood, but not required, by instances of this class. |
| `static UnmodifiableSet<java.lang.String>` | `UniqueCycleIdManager.optionalKeywords`             The set of keywords understood, but not required, by instances of this class. |
| `static UnmodifiableSet<java.lang.String>` | `PathIdManager.optionalKeywords`             The set of keywords understood, but not required, by instances of this class. |
| `static UnmodifiableSet<java.lang.String>` | `CycleIdManager.requiredKeywords`             The set of keywords required to be present in the `ManagerInput` objects supplied to instances of this class. |
| `static UnmodifiableSet<java.lang.String>` | `UniqueCycleIdManager.requiredKeywords`             The set of keywords required to be present in the `ManagerInput` objects supplied to instances of this class. |
| `static UnmodifiableSet<java.lang.String>` | `PathIdManager.requiredKeywords`             The set of keywords required to be present in the `ManagerInput` objects supplied to instances of this class. |

| Methods in sbrt.shell.mng.graph\_theory that return UnmodifiableSet | |
| --- | --- |
| `UnmodifiableSet<java.lang.String>` | `CycleIdManager.getOptionalKeys()`             Returns the set of keywords the provided `ManagerInput` object does not necessarily have to contain. |
| `UnmodifiableSet<java.lang.String>` | `UniqueCycleIdManager.getOptionalKeys()`             Returns the set of keywords the provided `ManagerInput` object does not necessarily have to contain. |
| `UnmodifiableSet<java.lang.String>` | `PathIdManager.getOptionalKeys()`             Returns the set of keywords the provided `ManagerInput` object does not necessarily have to contain. |
| `UnmodifiableSet<java.lang.String>` | `CycleIdManager.getRequiredKeys()`             Returns the set of keywords the provided `ManagerInput` object must contain. |
| `UnmodifiableSet<java.lang.String>` | `UniqueCycleIdManager.getRequiredKeys()`             Returns the set of keywords the provided `ManagerInput` object must contain. |
| `UnmodifiableSet<java.lang.String>` | `PathIdManager.getRequiredKeys()`             Returns the set of keywords the provided `ManagerInput` object must contain. |

Uses of UnmodifiableSet in sbrt.shell.mng.stat

| Fields in sbrt.shell.mng.stat declared as UnmodifiableSet | |
| --- | --- |
| `static UnmodifiableSet<java.lang.String>` | `MannWhitneyUManager.optionalKeywords`             The set of keywords understood, but not required, by this process manager. |
| `static UnmodifiableSet<java.lang.String>` | `KendallTauManager.optionalKeywords`             The set of keywords understood, but not required, by this process manager. |
| `static UnmodifiableSet<java.lang.String>` | `CorrelationManager.optionalKeywords`             The set of keywords understood, but not required, by this process manager. |
| `static UnmodifiableSet<java.lang.String>` | `MannWhitneyUManager.requiredKeywords`             The set of keywords required by this process manager. |
| `static UnmodifiableSet<java.lang.String>` | `KendallTauManager.requiredKeywords`             The set of keywords required by this process manager. |
| `static UnmodifiableSet<java.lang.String>` | `CorrelationManager.requiredKeywords`             The set of keywords required by this process manager. |

| Methods in sbrt.shell.mng.stat that return UnmodifiableSet | |
| --- | --- |
| `UnmodifiableSet<java.lang.String>` | `MannWhitneyUManager.getOptionalKeys()`             Returns the set of keywords understood, but not required, by this process manager. |
| `UnmodifiableSet<java.lang.String>` | `KendallTauManager.getOptionalKeys()`             Returns the set of keywords understood, but not required, by this process manager. |
| `UnmodifiableSet<java.lang.String>` | `CorrelationManager.getOptionalKeys()`             Returns the set of keywords understood, but not required, by this process manager. |
| `UnmodifiableSet<java.lang.String>` | `MannWhitneyUManager.getRequiredKeys()`             Returns the set of keywords required by this process manager. |
| `UnmodifiableSet<java.lang.String>` | `KendallTauManager.getRequiredKeys()`             Returns the set of keywords required by this process manager. |
| `UnmodifiableSet<java.lang.String>` | `CorrelationManager.getRequiredKeys()`             Returns the set of keywords required by this process manager. |

Uses of UnmodifiableSet in sbrt.shell.mng.utilities

| Fields in sbrt.shell.mng.utilities declared as UnmodifiableSet | |
| --- | --- |
| `static UnmodifiableSet<java.lang.String>` | `VariableParticipationManager.optionalKeywords`             The set of keywords understood, but not required, by instances of this class. |
| `static UnmodifiableSet<java.lang.String>` | `VectorComparisonManager.optionalKeywords`             The set of keywords understood, but not required, by this process manager. |
| `static UnmodifiableSet<java.lang.String>` | `IntervalComparisonManager.optionalKeywords`             The set of keywords understood, but not required, by this process manager. |
| `static UnmodifiableSet<java.lang.String>` | `VariableParticipationManager.requiredKeywords`             The set of keywords required to be present in the `ManagerInput` objects supplied to instances of this class. |
| `static UnmodifiableSet<java.lang.String>` | `VectorComparisonManager.requiredKeywords`             The set of keywords required by this process manager. |
| `static UnmodifiableSet<java.lang.String>` | `IntervalComparisonManager.requiredKeywords`             The set of keywords required by this process manager. |

| Methods in sbrt.shell.mng.utilities that return UnmodifiableSet | |
| --- | --- |
| `UnmodifiableSet<java.lang.String>` | `VariableParticipationManager.getOptionalKeys()`             Returns the set of keywords the provided `ManagerInput` object does not necessarily have to contain. |
| `UnmodifiableSet<java.lang.String>` | `VectorComparisonManager.getOptionalKeys()`             Returns the set of keywords understood, but not required, by this process manager. |
| `UnmodifiableSet<java.lang.String>` | `IntervalComparisonManager.getOptionalKeys()`             Returns the set of keywords understood, but not required, by this process manager. |
| `UnmodifiableSet<java.lang.String>` | `VariableParticipationManager.getRequiredKeys()`             Returns the set of keywords the provided `ManagerInput` object must contain. |
| `UnmodifiableSet<java.lang.String>` | `VectorComparisonManager.getRequiredKeys()`             Returns the set of keywords required by this process manager. |
| `UnmodifiableSet<java.lang.String>` | `IntervalComparisonManager.getRequiredKeys()`             Returns the set of keywords required by this process manager. |

---


|  |  |  |
| --- | --- | --- |
| **Overview** \| **Package** \| **Class** \| **Use** \| **Tree** \| **Deprecated** \| **Index** \| **Help** | |  |
| PREV   NEXT | **FRAMES**    **NO FRAMES**     **All Classes** |  |


---
